# The Impact of the Emotional Disposition of Healthcare Workers on the Expression of Adverse Events after Primary Vaccination against SARS-CoV-2

**DOI:** 10.3390/medicina60030383

**Published:** 2024-02-24

**Authors:** Jolanta Sauserienė, Danielius Serapinas, Ida Liseckienė, Aida Budrevičiūtė, Rūta Vaičiūnienė, Brigita Gradauskienė, Ema Tkačiovaitė, Sandra Sakalauskaitė, Leonas Valius

**Affiliations:** 1 Department of Family Medicine, Medical Academy, Lithuanian University of Health Sciences, LT-44307 Kaunas, Lithuania; danielius.serapinas@lsmu.lt (D.S.); ida.liseckiene@kaunoklinikos.lt (I.L.); aida.budreviciute@gmail.com (A.B.); leonas.valius@lsmu.lt (L.V.); 2 Department of Nephrology, Medical Academy, Lithuanian University of Health Sciences, LT-44307 Kaunas, Lithuania; 3 Department of Immunology and Allergology, Medical Academy, Lithuanian University of Health Sciences, LT-44307 Kaunas, Lithuaniasandra.sakalauskaite@lsmu.lt (S.S.); 4 Faculty of Medicine, Medical Academy, Lithuanian University of Health Sciences, LT-44307 Kaunas, Lithuania; ema.tkaciovaite@stud.lsmu.lt

**Keywords:** adverse events of vaccines, emotional disposition, Pfizer–BioNTech vaccine, vaccination against SARS-CoV-2, Lithuania

## Abstract

*Background and Objectives:* Vaccination is one means of SARS-CoV-2 prevention and control. However, despite the effectiveness of vaccination, adverse reactions continue to require vigilance and monitoring. The researchers emphasize the possibility that some of the reported side effects may be psychological in origin. Based on this hypothesis, the main goal of this study was to evaluate the emotional dispositions of healthcare workers who experienced emotions before vaccination and adverse reactions after vaccination. *Materials and Methods:* This study was conducted between February and May 2021 in the Kaunas Clinics of the University of Health Sciences. A total of 2117 employees of the clinic departments who were vaccinated with two doses of the Pfizer–BioNTech vaccine participated in this study. Statistical analysis was performed on the data using IBM SPSS Statistics^®^. *Results:* Most participants (74.5%) experienced systemic (including local) adverse events; 16.5% experienced only local adverse events, and 9.1% experienced no adverse events. The frequency of systemic (including local) adverse events reduced with increasing age (*p* < 0.05). The main emotions that participants experienced before vaccination were anxiety (37.88%) and happiness (39.02%). Systemic (including local) adverse events occurred 1.26 times more frequently in women than men (77.44% vs. 61.6%, *p* < 0.05), while local adverse events occurred 1.4 times more often in male participants than in female participants (21.39% vs. 15.27%, *p* < 0.05). Among the respondents who did not experience adverse events, the most common emotion felt was happiness (25.5%), and most of the participants who experienced systemic (including local) adverse events felt anxiety (42.6%). *Conclusions:* The information about vaccination and potential adverse events should be targeted at younger persons. It is recommended that women, more than men, should receive professional counseling from psychologists or psychotherapists. The public dissemination of positive messages about the benefits and safety of vaccines prior to a vaccination campaign may alleviate the tension or anxiety felt regarding potential adverse events. Healthcare specialists—both those who work directly with vaccines and those who do not—should maintain a positive psychological attitude towards vaccination, as this can increase patient satisfaction with the benefits of vaccines.

## 1. Introduction

Even at the onset of the COVID-19 pandemic, vaccination was outlined as the most effective way to control the disease. The COVID-19 vaccines were developed and deployed quickly with the hope of controlling the infection, which spread extremely quickly to all continents of the world [1]. However, this raised many questions about the safety, side effects, and efficacy of these vaccines [2]. After the first vaccines were approved, nearly 3.4 billion vaccine doses were administered in the 6 months that followed [3]. An unprecedented mass vaccination effort took place worldwide. The most commonly used vaccine is the BNT162b2 Pfizer–BioNTech mRNA vaccine, which is approved in 123 countries [4]. Studies showed that fever, fatigue, headache, and injection site pain are among the most common adverse events associated with this vaccine. Furthermore, serious adverse events include shoulder injury related to vaccine administration, right axillary lymphadenopathy, paroxysmal ventricular arrhythmia, right leg paresthesia, fatigue and headache [4]. The number of reported vaccine-related adverse events was also unprecedented. Some vaccines are even associated with myocarditis, including mRNA vaccines [5]. One study demonstrated that, after the administration of the first mRNA vaccine dose, 53.6% of participants experienced at least one adverse event, the majority of which were local reactions [6]. The risk of experiencing adverse events has become one of the main reasons for anti-SARS-CoV-2 vaccination hesitancy among healthcare professionals around the world [7]. Different strategies for choosing the best vaccine variant to devise vaccination protocols with the highest levels of efficiency and the lowest rates of adverse reactions have been analyzed [8]. Adverse reaction rates differ significantly from patient to patient. Multiple factors may be responsible for this, including individual psychological and genetic peculiarities [9]. There are two key types of factor in this instance: side effects that are directly attributable to the composition of the vaccine and those that are not vaccine related but, instead, represent nonspecific reactions to the process of becoming immunized [10]. Adverse effects are mainly due to hormonal and genetic gender differences: in females, for example, the expression of innate immunity features and interferon genes is stronger than in males [11]. There is an opinion that stronger inflammatory processes are triggered at this point, and even the suggestion that vaccination must be personalized by gender. In addition, age is an essential factor that negatively influences immunization [11,12].

One study which considered individuals 8 months after the administration of two doses of the anti-SARS-CoV-2 vaccine showed that the immune function among vaccinated participants was lower than among unvaccinated individuals [13]. This reduced immunity was as a result of several factors [13]:

- N1-methylpseudouridine is used as a substitute for uracil in the genetic code. Decreased cellular immunity can occur as a result of the activation of regulatory T cells, as induced by this modified protein.
- The cells and tissues that are primed to produce spike proteins are damaged by the newly generated antibodies of the spike protein. The organs of the immune system, such as the adrenal gland, may then be damaged after the vascular endothelial cells are damaged by the spike proteins in the bloodstream.
- The effect of neutralizing antibodies in preventing infection may be attenuated by infection-enhancing antibodies. This can cause antibody-dependent enhancement.

Some adverse reactions after vaccination possibly comprise a psychosomatic nocebo component that has been very poorly studied in individuals vaccinated against SARS-CoV-2 [14]. A study by Zhu et al. showed that, after receiving a physiological solution, 37% of participants in the placebo group reported adverse events. The most common of these adverse events were fatigue (17%), headache (13%), and pain at the injection site (9%) [15]. Personality type and psychological conditions may even be related to the antibody production level. For example, higher neuroticism scores were associated with a poorer antibody response after influenza vaccination, possibly because of blunted cortisol reactivity [16]. This means that negative feelings after vaccination may be caused not only by physiological issues but also by psychological aspects. Therefore, the goal of this study was to evaluate the emotional dispositions of healthcare workers that experienced emotions before vaccination, and adverse reactions after vaccination. The main research questions were as follows:

- How did adverse events differ in the different age groups of healthcare workers?
- What emotions were experienced before vaccination, and what adverse reactions were experienced after vaccination?
- What was the emotional disposition of study participants working in areas directly related to the COVID-19 pandemic?

## 2. Materials and Methods

### 2.1. Study Setting and Participants

This study was conducted between February and May 2021 in one of the largest medical institutions in Lithuania: the hospital of the Lithuanian University of Health Sciences Kaunas Clinics. The study began after the start of the vaccination of healthcare workers in Lithuania. A COVID-19 vaccination center was established in this hospital, and at first vaccinations were provided for medical workers. The first vaccine introduced to Lithuania was mRNA-based (Pfizer–BioNTech, Mainz, Germany), and hospital workers at high risk of SARS-CoV-2 infection were invited to be vaccinated, except those who had COVID-19 or exhibited medical contraindications. The weekly screening of hospital staff was conducted with confirmed PCR tests. These tests were also performed prior to vaccination. Thus, there was an extremely low likelihood that the study participants possessed specific antibodies against the SARS-CoV-2 virus before vaccination. This article presents the results of the second stage of the study. The participants of the second stage were all Kaunas Clinics employees who were vaccinated with two doses of the Pfizer–BioNTech vaccine within an interval of 21 days (±2), and who agreed to fill in a detailed questionnaire 3–5 weeks after receiving the second vaccine dose.

#### Study Instrument

Due to the emergence of a new field of research—the COVID-19 pandemic—and the lack of a standardized instrument suitable for the research design, the research team developed an anonymous questionnaire that was used in this study. In it, respondents were asked to indicate whether they experienced adverse events within 15 minutes of receiving the first and second vaccine doses by ticking the appropriate box (yes or no). Participants were also asked about the intensity of adverse events after leaving the vaccination center and on the first, second, third, fourth, and other days, and whether these effects required them to seek medical care at a healthcare facility after both doses of the vaccine. Questions were also asked about the health status of the participants, chronic diseases, medications used, work characteristics and their emotional states when attending vaccination. The intensity of the systemic and local adverse events listed in the standardized questionnaire, based on data from previous studies [17], was evaluated on a scale of 1 (very mild symptoms) to 5 (very severe symptoms). Then, the intensity of adverse events was assessed by asking participants to rate them separately on the first, second, third, fourth, and subsequent days after each vaccine dose. Participants were also encouraged to report symptoms not included in the list by recording them in the questionnaire. Adverse events after the vaccination were classified as local or systemic, and as mild (1–2 points) or severe (3–5 points). The participants also had to indicate one or more emotions (anxiety, fear, happiness and ambivalence) that they felt before the vaccination and rank their intensity from 1 (low intensity) to 5 (very high intensity). Finally, the demographic details of the participants were recorded using a questionnaire prompting them to indicate their age, gender, body mass index, and occupational and health status characteristics. A pilot study was conducted with 30 hospital employees to see if the new questionnaire was methodologically sound for use in research. The pilot study showed that the questions used had a Cronbach’s alpha of between 0.6 and 0.7, making the instrument suitable for total variance. A total of 5426 questionnaires were collected. Incomplete and damaged questionnaires were removed; however, if the vaccine recipients reported that they experienced adverse events or emotions but failed to rate their intensity, their questionnaires were included in the analysis, with unrated responses assigned as 1 (very mild) by default. Thus, the analysis included a total of 4181 completed questionnaires.

### 2.2. Data Processing

The chi-squared test or Fisher’s exact test was used to compare categorical data. The *z*-test was used to compare proportions between groups. The nonparametric Mann–Whitney criterion (for two groups) and the Kruskal–Wallis ranking criterion (for three or more groups) were used to compare quantitative data. Analysis was performed on the basis of the following categories: age group (<30, 30–46, 47–56 and ≥57 years according to the quartiles of 30, 47 and 57 years, respectively); gender; experience of adverse events (a binary variable: yes or no); use of medication and/or ability to work after vaccination (a binary variable: yes or no); experience of moderate adverse events; referral to a hospital due to adverse events (a binary variable: yes or no); and IgG status (a binary variable: positive or negative). A logistic regression analysis was performed, after which odds ratios (ORs) with 95% confidence intervals (95% CI) were calculated. Statistical significance was achieved at *p* < 0.05.

### 2.3. Ethics

This study was approved by the Kaunas Regional Biomedical Research Ethics Committee (permission No. BE-2-43, 5 February 2021).

## 3. Results

The majority of participants (74.5%) experienced systemic (including local) adverse events, while 16.5% experienced only local adverse events, and 9.1% of participants experienced no adverse events (Table 1).

By comparing different age groups, it became clear that, after vaccination, younger persons were more likely to experience adverse events. In short, as age increased, the frequency of systemic (including local) adverse events decreased (*p* < 0.05). The vast majority (88.55%) of vaccine recipients below 30 years of age experienced systemic (including local) adverse events, yet this percentage was lower (62.58%) among participants aged 57 and older. Conversely, local adverse events occurred more often with increasing age: 33.71% of all people who experienced local adverse events were 57 years and older. Only 9.07% of participants did not experience adverse events (Table 2).

The main emotions that participants experienced before vaccination were anxiety (37.88%) and happiness (39.02%). By comparing emotions between genders, it can be seen that male participants more often felt happiness and that female participants were more often anxious (*p* < 0.05). The minority of both genders felt ambivalence (5.15%). Moreover, women felt fear more often than men (15.04% vs. 6.44%, *p* < 0.001) (Table 3).

Systemic (including local) adverse events occurred 1.26 times more frequently in women than in men (77.44% vs. 61.6%, *p* < 0.05). Meanwhile, local adverse events occurred 1.4 times more often in male participants than in female participants (21.39% vs. 15.27%, *p* < 0.05). Furthermore, more men than women did not experience any adverse events (17.01% vs. 7.29%) (Table 4).

While researching the emotions of participants with different characteristics before and after vaccination, it was discovered that among all people who did not experience adverse events, the most common emotion felt was happiness (25.5%). Participants who experienced systemic (including local) adverse events mostly felt anxiety (42.6%). The least frequently experienced emotions were fear (1832 participants did not feel this emotion) and ambivalence (2008 participants did not experience this). People who felt fear more often experienced systemic (including local) adverse events (16.1%). However, participants who felt ambivalence mostly experienced local adverse events (5.5%) (Table 5).

Participants who had not worked with COVID-19 patients prior to vaccination reported anxiety as the most common of all four emotions (40.62%). A larger proportion of the participants who felt fear did not work with COVID-19 patients (80% vs. 20%), but this was statistically insignificant (*p* > 0.05). However, people who worked directly with COVID-19 patients before vaccination felt happiness (46.38%) (Table 6).

## 4. Discussion

Our study analyzed the psychological aspects of healthcare workers and the peculiarities of adverse reactions after mRNA vaccination against COVID-19. The key finding of this study was that adverse reactions, which were largely non-serious, were experienced by approximately 90% of participants. Life-threatening or severe allergic reactions were less frequent in younger individuals and females. Negative emotions and adverse reactions were stronger for women. An important finding of this study was that those who worked directly with COVID-19 patients had lower rates of negative emotions and higher rates of happiness before vaccination. 

The results showed that no life-threatening or severe allergic reactions were documented after the first vaccine dose in this large cohort of 2117 individuals. Symptoms including fever are reported in different studies with an incidence of up to 95% [18]. As previously reported [19], we also found that these reactions were more frequent in younger individuals and females. Lower rates of adverse reactions may be related to a reduced general immune response in advanced-age patients. This was also observed in previous studies [20]. We focused on an analysis of healthcare workers because they were on the front line during the COVID-19 pandemic. This group was particularly psychologically vulnerable because of the pandemic, with approximately 50% incidence rates of depression and anxiety across different medical practice settings [21]. We observed that the connection between negative emotions and adverse reactions was even stronger in females than in males. Generally, systemic adverse reactions in females were more frequent than in males. This finding coincides with other studies showing that females are more prone to adverse reactions after anti-SARS-CoV-2 vaccination [22]. One objective of our study was to evaluate how the emotions of healthcare workers before vaccination relate to adverse reaction rates. We found that half of our participants had negative emotions before vaccination (anxiety or fear).Negative emotions before vaccination were observed more frequently in females than in males. This may be explained by the fact that depression rates are generally higher in female populations than in male populations [23]. Some studies have also shown that psychological distress reported before vaccination is a risk factor for higher rates of post-vaccination fatigue and muscle pain [24]. On the other hand, healthcare workers with negative emotions before vaccination could have worse general psychological states. Other studies also support these causal links [25]. Thus, the mechanisms explaining our results could be complex. Both factors for anxiousness are important: the general psychological state of the person and additional worries related to the information about vaccination, which sometimes seems controversial. Some studies have shown that healthcare workers with depression or suicidal thoughts experienced more frequent systemic adverse reactions after vaccination [26]. One possible explanation for this may be the fact that depression is associated with general low-grade systemic inflammation [27]. Even data collected from healthy individuals show that, after mRNA vaccination against COVID-19, the markers of systemic inflammation (IL-16) may remain elevated in the bloodstream for some months [28]. When analyzing healthcare workers by their roles at work, we observed that those who work directly with COVID-19 patients had lower rates of negative emotion and higher rates of happiness before vaccination. This is a very important issue, showing that this specific group of healthcare workers was waiting for vaccination and was less hesitant or worried before receiving the vaccine. This can be explained by studies which show that front-line workers face a great deal of distress because of the risk of being infected with SARS-CoV-2 [29,30]. Overall, substantial negative mental/psychological health outcomes are events to which healthcare workers aiding in COVID-19 control are especially vulnerable. These can include stress-related symptoms, symptoms of depression, anxiety, and insomnia [31]. Moreover, front-line healthcare workers were at increased risk of reporting a positive COVID-19 test when compared with the general community [32], and a decrease in the acceptance of the vaccine against COVID-19 among these healthcare workers was associated with vaccine hesitancy [33]. The main reasons for vaccine hesitancy include the following: poor health literacy; safety concerns, especially among the elderly and people with various preexisting comorbidities; confusion over protection levels; doubts about the efficacies of the available vaccines against emerging SARS-CoV-2 variants; the perceived risk and fears; a lack of awareness about the SARS-CoV-2 virus; misinformation or a lack of accurate knowledge about vaccines; anti-vaccine myths and confusing messages about the severe side effects of certain vaccines; under-representation in health research; a general mistrust and suspicion of medical companies exacerbated by the lack of legal liability of vaccine manufacturers; and political and economic intentions that were perceived to be driving the pandemic or vaccine development [34]. Another study showed that vaccine hesitancy among healthcare workers was predominantly caused by a lack of trust in the anti-SARS-CoV-2 vaccines, with 76% of respondents noting doubts regarding vaccine efficacy and 85% pointing to their fear of side effects [35]. These concerns are perhaps buoyed by a lack of comprehensive, trustworthy data and the media controversy surrounding this issue. This is highlighted in one study by the significant number of participants observing that they possess little (78%) or conflicting (69%) information about vaccines [35]. COVID-19 vaccine hesitancy may be one reason for stress-related adverse reactions after vaccination [34]. The huge efforts of the healthcare workforce during the COVID-19 pandemic had a profound impact on their mental health, and prioritizing the psychological well-being of clinicians will be vital in the future. This will not only support the health of clinicians, but will also help to ensure that their ability to face the challenges posed by the COVID-19 pandemic—and any future public health challenges—remains strong. Therefore, before vaccination, not only should we seek to obtain complete information on patients’ previous medical and family history, but we should also evaluate their psychological state. A personalized approach, along with a psychological assessment, should be applied before vaccination. The intensification of health-promoting programs is clearly necessary, and psychosocial perspectives on stress reduction might be included in this process so that individuals with vaccine hesitancy can be more informed [36].

Our future work will be focused on psychological assessments. Here, interventions before vaccination will be critical, as will the design of pragmatic trials to assess the adoption of such strategies in a diverse range of practice settings and among a wide range of healthcare professionals.

## 5. Conclusions

The majority of participants experienced systemic, including local, adverse events, and nearly one-fifth reported experiencing only local adverse events. Younger participants experienced adverse events post-vaccination more frequently than older participants; therefore, information about vaccines and their potential adverse events should be targeted at younger age groups. Male participants reported feeling happiness more often than female participants; anxiety and fear were reported more often by females than males. Female participants also reported experiencing more negative emotions (anxiety, fear) pre-vaccination and adverse events post-vaccination; therefore, professional (psychologist and psychotherapist) counselling services should be provided for females more urgently than for males. More than a quarter of the participants who experienced no adverse events post-vaccination reported feeling happiness, while over half of those who did experience adverse events reported feeling anxiety. Based on this, it is important to publicly disseminate positive messages about the benefits and safety of vaccines prior to a vaccination campaign so as to alleviate any tension or anxiety about potential vaccine-related adverse events. Nearly half of the healthcare workers who worked with COVID-19 patients directly prior to vaccination expressed feeling happiness. Over half of those not working directly with COVID-19 patients reported feeling anxiety; therefore, it is crucial to improve the positive psychological attitude of healthcare workers towards vaccination. It is crucial that positive psychological attitudes are encouraged and that vaccination and immunization programs are integrated into professional healthcare studies and professional development courses. The participants rarely reported feeling fearful, but because almost half reported having anxiety, psychological attitudes, professional counselling, and public information campaigns can be effective health literacy tools for increasing the positive outlook of individuals prior to vaccination and reducing the risk of subsequent attitude-dependent adverse events post-vaccination. 

## Figures and Tables

**Table 1:** Characteristics of the study participants and their experience of adverse events.

Characteristic (*n* = 2117)	Value
Age, mean (SD), years	47.06 (14.2)
Gender	
Female Male	1729 (81.7) 388 (18.3)
Did not experience AE	192 (9.1)
Only local AE	347 (16.4)
Systemic (including local) AE	1578 (74.5)

AE—adverse events.

**Table 2:** Incidence of adverse events among different age groups.

Age Group in Years	Total *n*	Did Not Experience AE *n (%)*	Experienced Local AE *n (%)*	Experienced Systemic AE *n (%)*	*p*
<30	374	4 (1.07)	39 (10.43)	331 (88.55)	<0.001
30–46	559	27 (4.83)	95 (16.99)	437 (78.18)	
47–56	556	43 (7.73)	96 (17.27)	417 (75.00)	
≥57	628	118 (18.79)	117 (18.63)	393 (62.58)	
Total	2117	192 (9.07)	347 (16.39)	1578 (74.54)	

AE—adverse events.

**Table 3:** Participants distributed according to emotions before vaccination.

Emotion	Total *n*	Anxiety *n* (%)	Fear *n* (%)	Happiness *n* (%)	Ambivalence *n* (%)
Total	2117	802 (37.88)	285 (13.46)	826 (39.02)	109 (5.15)
Male	388	79 (20.36)	25 (6.44)	199 (51.29)	22 (5.67)
Female	1729	723 (41.82)	260 (15.04)	627 (36.26)	87 (5.03)
*p* ^1^		<0.001	<0.001	<0.001	0.607

^1^ Male versus female.

**Table 4:** Characteristics of participants by gender and AE.

Characteristic	Did Not Experience AE *n* = 192	Experienced Local AE *n* = 347	Experienced Systemic AE *n* = 1578	*p*
Female (*n* = 1729)	126 (7.29)	264 (15.27)	1339 (77.44)	<0.001
Male (*n* = 388)	66 (17.01)	83 (21.39)	239 (61.60)	
Age, mean (SD), years	58.27 (10.911)	49.67 (13.64)	45.12 (14.00)	<0.001

Values are numbers (percentages) unless otherwise indicated. AE—adverse events.

**Table 5:** Characteristics of participants with different emotions before vaccination and adverse events after vaccination.

Emotion		Did Not Experience AE *n* = 192	Experienced Local AE *n* = 347	Experienced Systemic AE *n* = 1578	*p*
Anxiety	No (0 points) (*n* = 1315)	165 (85.9)	244 (70.3)	906 (57.4)	<0.001
	Yes (1–5 points) (*n* = 802)	27 (14.1)	103 (29.7)	672 (42.6)	
Fear	No (0 points) (*n* = 1832)	186 (96.9)	322 (92.8)	1324 (83.9)	<0.001
	Yes (1–5 points) (*n* = 285)	6 (3.1)	25 (7.2)	254 (16.1)	
Happiness	No (0 points) (*n* = 1291)	143 (74.5)	213 (61.4)	935 (59.3)	<0.001
	Yes (1–5 points) (*n* = 826)	49 (25.5)	134 (38.6)	643 (40.7)	
Ambivalence	No (0 points) (*n* = 2008)	185 (96.4)	328 (94.5)	1495 (94.7)	0.605
	Yes (1–5 points) (*n* = 109)	7 (3.6)	19 (5.5)	83 (5.3)	

AE—adverse events.

**Table 6:** Participants’ emotions depending on whether they directly worked with COVID-19 patients or not.

Emotion		Did Not Work Directly	Worked Directly	*p*
Anxiety	No (0 points) (*n* = 1277)	1001 (62.0)	276 (60.7)	0.598
	Yes (1–5 points) (*n* = 792)	613 (38.0)	179 (39.3)	
Fear	No (0 points) (*n* = 1789)	1390 (86.1)	399 (87.7)	0.387
	Yes (1–5 points) (*n* = 280)	224 (13.9)	56 (12.3)	
Happiness	No (0 points) (*n* = 1255)	1024 (63.4)	231 (50.8)	<0.001
	Yes (1–5 points) (*n* = 814)	590 (36.6)	224 (49.2)	
Ambivalence	No (0 points) (*n* = 1963)	1532 (94.9)	431 (94.7)	0.868
	Yes (1–5 points) (*n* = 106)	82 (5.1)	24 (5.3)	

## Data Availability

The data are only available upon request due to restrictions, e.g., privacy and ethical restrictions. The data presented in this study are available upon request from the corresponding author.
